# Analysis of medical service use of knee osteoarthritis and knee meniscal and ligament injuries in Korea: a cross-sectional study of national patient sample data

**DOI:** 10.1186/s12891-017-1795-7

**Published:** 2017-11-10

**Authors:** Chang Yong Suh, Yoon Jae Lee, Joon-Shik Shin, Jinho Lee, Me-riong Kim, Wonil Koh, Yun-Yeop Cha, Byung-Cheul Shin, Eui-Hyoung Hwang, Kristin Suhr, Mia Kim, In-Hyuk Ha

**Affiliations:** 1 Jaseng Spine and Joint Research Institute, Jaseng Medical Foundation, 858 Eonju-ro, Gangnam-gu, Seoul, Republic of Korea; 2 Department of Applied Korean Medicine, College of Korean Medicine, Graduate School, Kyung Hee University, Dongdaemun-gu, Seoul, Republic of Korea; 3 Department of Rehabilitation Medicine of Korean Medicine, College of Korean Medicine, Sangji University, Wonju-si, Gangwon-do Republic of Korea; 4 Spine & Joint Center, Pusan National University Korean Medicine Hospital, Yangsan-si, Gyeongsangnam-do Republic of Korea; 5 Department of Korean Rehabilitation Medicine, School of Korean Medicine, Pusan National University, Yangsan-si, Gyeongsangnam-do Republic of Korea; 6 Prevention Sciences, Rollins School of Public Health, Emory University, Atlanta, GA USA; 7 Department of Cardiovascular and Neurological Diseases (Stroke Center), College of Korean Medicine, Kyung Hee University, Seoul, Republic of Korea

**Keywords:** Knee osteoarthritis, Knee meniscal and ligament injury, Medical service use, Usual care, Korean Health Insurance Review and Assessment Service-National Patient Sample (HIRA-NPS) data

## Abstract

**Background:**

Osteoarthritis (OA) and meniscal and ligament injuries of the knee are the two most common knee disorders in Korea. The aim of this study was to analyze the demographic characteristics, medical service use and related costs for these disorders, and the results are expected to help inform practitioners, researchers, and policy-makers.

**Methods:**

The present study aimed to evaluate incidence and patient characteristics, and to assess current medical service use, usual care, and medical expenses of knee disorders by analyzing 2014 national patient sample data from the Korean Health Insurance Review and Assessment Service. Data was extracted using 3% stratified sampling from all Korea national health insurance claims submitted in 2014, and analyzed. Usual care for M17 knee osteoarthritis and S83 knee meniscal and ligament injury codes of the International Classification of Diseases, 10th revision (ICD-10) were determined by investigating total number of patients, sociodemographic characteristics, days in care, number of visits, and expenses.

**Results:**

Knee OA showed the highest incidence in females aged ≥60 years, whereas meniscal and ligament injuries of the knee were most prevalent among patients aged <20 years and young adults. Total inpatient care expenses exceeded the cost of ambulatory care for both disorders. Ambulatory care was mainly provided at primary care clinics, with 90% of these visits made to orthopedic specialists. Medical expenses for knee OA and meniscal and ligament injuries were largely due to procedures/surgeries and injections, and procedures/surgeries and hospitalizations, respectively. Total replacement arthroplasty was the most commonly performed surgery for knee OA, while meniscectomy and cruciate ligament reconstruction were the most often performed surgeries for meniscal and ligament injuries. Intra-articular injection rates were 55% in knee OA patients and 3% in meniscal and ligament injury patients. Aceclofenac, diclofenac, and tramadol were the most frequently prescribed analgesics.

**Conclusions:**

The current findings may be used as basic data for establishing medical policies and can benefit researchers and clinicians in recognizing trends and patterns of treatment for knee disorders.

**Electronic supplementary material:**

The online version of this article (10.1186/s12891-017-1795-7) contains supplementary material, which is available to authorized users.

## Background

Knee osteoarthritis (OA) is one of the most common disorders in the U.S., and symptomatic OA prevalence was found to be as high as 10% in men and 13% in women aged ≥60 [1]. Similarly, symptomatic knee OA was shown to affect 9.3% and 28.5% of Korean men and women aged ≥50, respectively, with a steep increase in prevalence in older populations [2]. Meanwhile, knee meniscal injury patients tend to be younger than knee OA patients, and incidence was reported as 3–5% in the U.S. [3] and 10.6% in Korean populations [4]. According to the U.S. Medical Expenditure Panel Survey, insurance coverage and out-of-pocket expenses for knee OA amounted to 185 billion U.S. dollars, denoting its significant socioeconomic impact [5]. In addition, arthroscopic partial meniscectomy is one of the most frequently performed orthopedic surgeries in the U.S., with 700,000 new cases performed each year, and estimates for direct annual medical costs were put at 4 billion U.S. dollars [6].

The epidemiology and pathology of knee OA and meniscal injury differ substantially regarding their onset, age, and etiology. Population-wide studies have purported that incidence estimates of knee meniscal injury are highest in adolescent and young men [7], stating that these populations are at higher risk of injury as they are more likely to engage in competitive sports such as ball games, while older adults are at lower risk of injury as they are more likely to participate in non-competitive sports such as walking, jogging and swimming [8]. On the other hand, knee OA prevalence increases rapidly after the age of 50, which is especially pronounced in post-menopausal women due to the effect of hormonal imbalance on cartilaginous tissue [9]. Although the epidemiology of knee osteoarthritis and injury varies considerably, their etiology is often viewed to share certain traits; injury of the anterior cruciate ligament (ACL) or the meniscus may incur joint instability and damage the cartilage surface, leading to chronic disability and potential knee OA [10, 11].

The National Health Insurance Service (NHIS) in Korea covers 47 million out of 51 million South Korean residents. Claims data are generated when a medical institution provides medical services to a patient and applies for reimbursement from the NHIS, and therefore contain information on the patient and medical institution, a complete list of insured medical services that were provided (e.g., treatment, examinations, and prescriptions), and their related costs. The two highest frequency knee disorders, knee OA and knee meniscal and ligament injuries, were comprehensively analyzed for patient characteristics and expenditure (i.e., surgery, hospitalization, physical therapy and medication costs) to the aim of providing basic information to policy-makers and practitioners in Korea. Moreover, given that standard care based on evidence and the usual care provided in real world settings frequently digress and that by country and culture, the implications of current reports on high-frequency medical service use (i.e., pharmacological, nonpharmacological, and surgical interventions) hold relevance at an international level also through illustration of concurrent clinical practice in knee disorders.

The aim of this study was to provide preliminary data towards establishing a basic guideline for general medical care based on the prevalence, current use of medical services, and costs of knee OA and meniscal and ligament injuries from the 2014 claims data submitted to the Korea Health Insurance Review and Assessment Service (HIRA). This analysis may be used to assist policymakers, practitioners, and researchers by providing a window into the most common treatments for knee conditions.

## Methods

### Data and subjects

The 2014 Health Insurance Review and Assessment Service-National Patient Sample (HIRA-NPS) data were analyzed. Claims data are generated when medical institutions apply for reimbursement of medical costs partially covered by Korea National Health Insurance. The data include medical record details (e.g., treatments, procedures, examinations, and prescriptions), diagnosis codes, co-payment paid by the patient and the insurance benefit paid by Korea National Health Insurance, patient demographics (e.g., age and sex), and information on the service provider (medical institution).

Provided yearly for research purposes, the HIRA-NPS datasets are extracted from the raw claims database using random sampling, stratified according to age groups and sex, with removal of identifying personal and institutional information. Each dataset consists of claims records for the corresponding year. Regardless of inpatient/outpatient status, 3% of the total patient population for each year was sampled and extracted, which approximates to 1.4 million patients. As each individual patient is coded with a unique identifier which is randomly generated and not privacy-sensitive, multiple visits of individual patients are easily traceable [12]. The current study chose to analyze the total number of patients by treatment type as opposed to total number of visits to the aim of illustrating individual medical service use in this patient population. Upon analysis, the results were presented both as inpatient and outpatient groups separately, and in total. The number of in- and outpatients indicate the number of patients who were hospitalized and those who visited the outpatient department one or more times for knee disorder-related diagnosis codes during the corresponding year, respectively. Multiple hospitalizations or outpatient visits of individual patients were viewed as duplicates and disregarded. Total counts for medical services were similarly tallied as the number of patients who used each respective service and not the number of treatment sessions. The actual parameters of the South Korean population may therefore be estimated from the current sample data statistics by multiplying the weighting value of 33.3 as the database is generated from 3% stratified sampling of total patients. In the present study, the statistical weight was not applied as the weighting was identical across all data and therefore did not affect the interpretation of results.

### Knee OA and knee meniscal and ligament injuries

Knee disorders were classified as M17 (arthrosis of the knee), S83 (dislocation, sprain, and strain of joints and ligaments of the knee), M22 (disorders of the patella) and M23 (internal derangement of the knee), according to the International Statistical Classification of Diseases and Related Health Problems, 10th revision (ICD-10). The number of individuals with knee disorder codes and radiological imaging of the knee were 48,321 with M17, 3087 with M22, 7103 with M23, and 19,136 with S83 in the 2014 dataset (Additional file 1: Table S1). Only those coded as M17 and S83 were included in this study as relatively few individuals were diagnosed with M22 and M23. A total of 48,000 outpatients and 3084 inpatients were diagnosed and treated under M17, and 18,540 outpatients and 2434 inpatients were diagnosed and treated under S83. Duplicate cases existed when a patient received both ambulatory and hospital care under the same code; thus, the total number of patients was smaller than the numerical sum of inpatients and outpatients. Through literature review and author discussion, M17 was diagnosed as knee OA [13–15] and S83 as knee meniscal and ligament injury [16].

### Analysis

The demographic characteristics of knee disorder patients as classified using ICD-10 diagnostic codes were examined. Age groups were divided into 10-year intervals: less than 20 years, 20–29 years, 30–39 years, and so on. Each patient was also classified by type of insurance as being eligible for either National Health Insurance or Medicaid. Medical institutions were categorized as primary care clinics, hospitals, general hospitals, tertiary hospitals, long-term care hospitals, or public health centers. The specialty of the attending physician was also assessed. The total treatment expenses were divided and analyzed according to the medical service codes designated by the Korean Ministry of Health and Welfare (i.e., costs per visit (consultation), hospitalization, medication, injection, anesthesia, physiotherapy, psychotherapy, procedure/surgery, examination, and radiographic evaluation/intervention). Total expense was defined as the total costs incurred for healthcare of the insured patient at medical institutions, which equaled the sum of benefit paid by the National Health Insurance Service and co-payment expenses paid by patients, of which the reimbursement amount was reviewed and determined by HIRA.

Details on the use of surgeries, injections, physiotherapy, and analgesics were investigated according to the corresponding code count; the relevant codes for these services are summarized in Additional file 2: Table S2. When assessing surgical patterns, the procedure codes were excluded from the procedure/surgery analysis because surgery codes were always accompanied by collateral procedure codes. Surgery codes that are not limited to the knee such as joint excision, osteochondral autograft transplantation, osteotomy and internal fixation, and subcutaneous tenotomy were also excluded.

Injection use was investigated according to the administration route, with intra-articular, subcutaneous, and intramuscular injections being of particular interest. Although intravenous, continuous intravenous, and perineural injections were often administered for knee disorders in clinical settings, the authors concluded that these injection types were generally not directly related to knee disorder treatment or for anti-nociceptive purposes, and these codes were accordingly excluded.

The most frequently applied physiotherapies are arranged in order of their prescription frequencies. Analgesics prescribed for inpatient and outpatient use were classified in order of prescription frequency, according to the 5th Anatomical Therapeutic Chemical (ATC) Classification System levels. Narcotic and non-narcotic substances were categorized as previously reported by the Korean National Evidence-based Health Care Collaborating Agency [17]. ATC codes were developed by the WHO Collaborating Centre for Drug Statistics Methodology in 1976 [18]. The 1st, 2nd, 3rd, 4th, and 5th ATC code levels indicate the anatomical target groups, therapeutic groups, therapeutic/pharmacologic subgroups, chemical/therapeutic/pharmacologic subgroups, and chemical substances, respectively, for the systematic classification of drug substances [19].

Most 5th ATC level drug substances have corresponding chemical names, with a few exceptions such as acetaminophen and paracetamol. Although this study used 5th ATC level terms to standardize nomenclature, four analgesics (7 in Additional file 3: Table S3) did not have corresponding 5th level codes, and in those instances, chemical names were used instead. Drug prescriptions were identified using the relevant claims data, regardless of dose.

### Statistical analysis

The demographic characteristics and medical details regarding surgeries, injections, physiotherapy, and drug prescriptions of patients with knee OA and meniscal and ligament injury are presented as frequencies and percentages (%) following frequency analyses. Patient percentages were determined using the total number of patients in each relevant diagnosis group as the denominator, and medical expense percentages were similarly calculated for total expenses, per-patient expenses, and costs per service using the total treatment expense for knee OA and knee meniscal and ligament injury as the denominator, respectively. Statistical analyses were performed using SAS, ver. 9.3 package (SAS Institute Inc., Cary, NC, USA).

## Results

### Current use of medical treatment for knee OA and knee meniscal and ligament injuries

The total number of patients, total expenses, per-patient expenses, average days of care, and average number of visits were higher for patients with knee OA than for those with knee meniscal and ligament injuries, regardless of whether the patients were treated as inpatients or outpatients. Although the total number of inpatients was smaller than the total number of outpatients, the total expenses for inpatients were higher in both types of knee disorders. The per-patient expense for inpatients was approximately 20 times higher than that for outpatients, irrespective of the type of knee disorder. However, the average number of days of care and number of visits were more than three times higher for hospitalized patients than for those receiving ambulatory care (Table 1).

**Table 1:** General medical service use and expenses for knee osteoarthritis and knee meniscal and ligament injury in Korea

Visit type	Diagnostic groups	Number of patients	Total expense^a^	Expense-per-patient^a^	Days of treatment^b^	Number of visits^c^
Total	Knee OA	48,321	24,526,738,820	507,579.3	11.0	9.1
	Knee meniscal and ligament injury	19,136	5,800,760,680	303,133.4	5.8	4.7
Outpatients	Knee OA	48,000	10,758,908,490	224,143.9	8.7	7.8
	Knee meniscal and ligament injury	18,540	1,660,439,710	89,559.9	3.4	3.2
Inpatients	Knee OA	3084	13,767,830,330	4,464,277.0	36.4	22.2
	Knee meniscal and ligament injury	2434	4,140,320,970	1,701,035.7	20.1	12.3

*OA* Osteoarthritis

^a^Displayed in KRW; 1 USD = 1104 KRW (as of September 30th, 2016)

^b^The total days of treatment indicated in the claims statement including drug prescription days without medical treatment

^c^The number of outpatient visits or the number of inpatient care days of the patient indicated in the claims statement

### Sociodemographic characteristics of knee OA and knee meniscal and ligament injury patients

The majority of patients with OA of the knee and knee meniscal and ligament injury received ambulatory care with approximately 6% of knee OA patients and 12% of meniscal and ligament injury patients hospitalized. The incidence of knee OA was positively correlated with age, showing a steep increase after the age of 50 years. However, the number of knee meniscal and ligament injury patients was highest in age < 20 years, with the numbers remaining constant from age 20 to 60 years, and declining thereafter. The age distribution was similar for both inpatients and outpatients with knee OA. On the other hand, the percentage of knee meniscal and ligament injury in patients aged <20 years who were hospitalized was approximately half the number of those receiving outpatient care. Knee OA was found to be more prevalent in women than in men, as was clearly evidenced by the fact that 82% percent of inpatients were women. On the contrary, the incidence of knee meniscal and ligament injuries was slightly higher in men than in women.

Almost all knee disorder patients were covered by National Health Insurance, and the treatment patterns did not differ substantially between inpatients and outpatients. About 70% of all patients visited primary care clinics, while the rest visited hospitals, general hospitals, and tertiary hospitals (listed in order of decreasing frequency of use). Notably, more than half of all knee OA and meniscal and ligament injury inpatients received medical care in hospitals instead of more accessible primary care clinics, which differed from the pattern seen in outpatients, and the percentage of general, tertiary, and long-term care hospitals increased substantially in inpatients. However, overall usage of medical institutions did not differ greatly between knee OA and meniscal and ligament injury patients. Approximately 90% of all patients, regardless of the type of disorder or whether they were receiving inpatient or outpatient care, sought medical care from an orthopedics department. Apart from orthopedics, knee OA patients were treated in anesthesiology, general surgery, and neurosurgery departments, and knee meniscal and ligament injury patients were treated in general surgery, neurosurgery, and emergency medicine (EM) departments. Other than orthopedics, inpatients were often treated in specialty departments such as rehabilitative medicine and general surgery, whereas radiology, EM, and general practice departments were utilized for outpatients (Table 2).

**Table 2:** Sociodemographic characteristics of knee osteoarthritis and knee meniscal and ligament injury patients

Characteristics		Total				Inpatient				Outpatient			
		Knee OA		Knee meniscal and ligament injury		Knee OA		Knee meniscal and ligament injury		Knee OA		Knee meniscal and ligament injury	
		*N* = 48,321	%	*N* = 19,136	%	*N* = 3084	%	*N* = 2434	%	*N* = 48,000	%	*N* = 18,540	%
Age (years)	< 20	243	0.50	4290	22.42	1	0.03	272	11.18	242	0.50	4252	22.93
	20~29	457	0.95	2665	13.93	11	0.36	411	16.89	450	0.94	2592	13.98
	30~39	890	1.84	2488	13.00	38	1.23	359	14.75	877	1.83	2415	13.03
	40~49	3812	7.89	3131	16.36	199	6.45	470	19.31	3758	7.83	3032	16.35
	50~59	12,856	26.61	3542	18.51	772	25.03	539	22.14	12,736	26.53	3387	18.27
	60~69	14,790	30.61	1865	9.75	960	31.13	251	10.31	14,729	30.69	1777	9.58
	≥ 70	15,273	31.61	1155	6.04	1103	35.77	132	5.42	15,208	31.68	1085	5.85
Sex	Male	13,141	27.20	10,445	54.58	546	17.70	1438	59.08	13,054	27.20	10,171	54.86
	Female	35,180	72.80	8691	45.42	2538	82.30	996	40.92	34,946	72.80	8369	45.14
Insurance type^a^	NHI	45,183	93.51	18,483	96.59	2853	92.51	2356	96.80	44,879	93.50	17,912	96.61
	MD	3234	6.69	651	3.40	230	7.46	74	3.04	3215	6.70	625	3.37
	VH	135	0.28	21	0.11	11	0.36	6	0.25	133	0.28	19	0.10
Medical institution type^b^	Clinic	36,731	76.01	13,343	69.73	592	19.20	590	24.24	36,570	76.19	13,115	70.74
	Hospital	12,925	26.75	4814	25.16	1816	58.88	1263	51.89	12,616	26.28	4452	24.01
	GH	5371	11.12	2053	10.73	646	20.95	569	23.38	5251	10.94	1872	10.10
	TH	1801	3.73	437	2.28	243	7.88	129	5.30	1774	3.70	408	2.20
	LCH	621	1.29	85	0.44	176	5.71	25	1.03	457	0.95	65	0.35
	PHC	407	0.84	21	0.11	2	0.06	–	–	407	0.85	21	0.11
	KMH	126	0.26	59	0.31	80	2.59	34	1.40	50	0.10	26	0.14
Medical specialty^c^	OS	43,654	90.34	17,056	89.13	2852	92.48	2304	94.66	43,379	90.37	16,486	88.92
	AN	3108	6.43	300	1.57	32	1.04	11	0.45	3083	6.42	293	1.58
	GS	2350	4.86	588	3.07	91	2.95	56	2.30	2281	4.75	554	2.99
	NS	1957	4.05	493	2.58	76	2.46	27	1.11	1899	3.96	470	2.54
	IM	1888	3.91	428	2.24	50	1.62	23	0.94	1846	3.85	410	2.21
	RM	1532	3.17	354	1.85	166	5.38	53	2.18	1408	2.93	323	1.74
	FM	1656	3.43	220	1.15	110	3.57	26	1.07	1563	3.26	195	1.05
	RD	258	0.53	295	1.54	1	0.03	1	0.04				
	ER	101	0.21	451	2.36	1	0.03	4	0.16				
	GP	326	0.67	19	0.10	1	0.03	1	0.04				
	Other^d^	470	0.97	93	0.49								

*OA* Osteoarthritis

^a^NHI, National Health Insurance; MD, Medicaid; VH, Veteran Healthcare

^b^GH, General Hospital; TH, Tertiary Hospital; LCH, Long-term Care Hospital; PHC, Public Health Center; KMH, Korean Medicine Hospital

^c^OS, Orthopedic surgery; AN, Anesthesiology; GS, General Surgery; NS, Neurosurgery; IM, Internal Medicine; RM, Rehabilitation Medicine; FM, Family Medicine; RD, Radiology; EM, Emergency Medicine; GP, General Physician

^d^Including Neurology, Thoracic and Cardiovascular Surgery, Pediatrics, Obstetrics and Gynecology, Urology, and Neuropsychiatry

### Distribution of medical expenses in knee OA and knee meniscal and ligament injuries

Medical expenses for knee OA and knee meniscal and ligament injury cases were classified into 10 categories: costs of visits (consultations), hospitalizations, medications, injections, anesthesia, physiotherapy, psychotherapy, procedures/surgeries, examinations, and radiologic evaluations/interventions. The categories reflecting the bulk of total expenses for each diagnostic group were, in decreasing order, procedures/surgeries, injections, visits (consultations), and hospitalizations for knee OA patients, and procedures/surgeries, hospitalizations, visits (consultations), and injections for knee meniscal and ligament injury cases. Procedures/surgeries constituted ≥30% of total expenses in both knee disorder categories, and injection costs comprised nearly 20% of the total expenses for knee OA patients, ranking 2nd in total costs, but only 6% of the total expenses for meniscal and ligament injury patients. More than 99% of all patients, irrespective of specific knee disorder, paid for cost of visits (consultations), and this category represented the third highest percentage of total costs for patients with either disorder. In addition, hospitalization costs also comprised a considerable portion of total medical expenses. In particular, hospitalization costs represented almost 20% (second highest percentage) of the total expenses for knee meniscal and ligament injury patients. Although more than 13% of all patients were prescribed medications, total medication costs were <2% of the total expenses for patients with either knee disorder. Similarly, ≥53% of all patients underwent physiotherapy, but this treatment cost represented only 6% of total expenses. The other subcategories (i.e., radiographic evaluations/interventions, examinations, and anesthesia) took up <6% of all expenses, and psychotherapy was rarely performed. Despite its relatively low proportion out of total costs, radiographic evaluations/interventions were widely applied to 98% of knee OA patients and 94% of meniscal and ligament injury patients (Table 3).

**Table 3:** Distribution of medical expenditure in knee osteoarthritis and knee meniscal and ligament injury

Classification	Knee OA					Knee meniscal and ligament injury				
	Case (n)		Cost^a^		Per-case cost^a^	Case (n)		Cost^a^		Per-case cost^a^
	*N* = 48,321	%	Total	%		*N* = 19,136	%	Total	%	
Procedure/surgery	5403	11.18	7,593,254,872	31.41	1,405,377.5	4555	23.80	1,887,655,557	34.31	414,414.0
Injection	37,050	76.67	4,732,291,088	19.58	127,727.2	8315	43.45	365,245,211	6.64	43,926.1
Outpatient visit (consultation)	48,283	99.92	4,021,230,425	16.64	83,284.6	19,084	99.73	715,896,835	13.01	37,512.9
Hospitalization	3033	6.28	2,757,254,594	11.41	909,084.9	2403	12.56	1,186,629,123	21.57	493,811.5
Physiotherapy	25,667	53.12	1,337,965,293	5.54	52,127.8	11,048	57.73	334,564,005	6.08	30,282.8
Radiographic evaluation/intervention	47,232	97.75	1,277,960,897	5.29	27,057.1	17,919	93.64	323,895,267	5.89	18,075.5
Examination	10,310	21.34	1,144,231,241	4.73	110,982.7	3351	17.51	357,995,050	6.51	106,832.3
Anesthesia	8576	17.75	907,924,433	3.76	105,868.1	1829	9.56	224,006,652	4.07	122,474.9
Medication	6327	13.09	399,489,786	1.65	63,140.5	3043	15.90	105,707,519	1.92	34,737.9
Psychotherapy	21	0.04	674,087	0.00	32,099.4	1	0.01	12,579	0.00	12,579.0

*OA* Osteoarthritis

^a^Displayed in KRW; 1 USD = 1104 KRW (as of September 30th, 2016)

### Usual care of knee OA and knee meniscal and ligament injuries, excluding medications

Frequently used surgeries, injections, and physiotherapies were investigated to determine usual practice patterns. Replacement arthroplasty was the most frequently performed surgery for knee OA patients, and cruciate ligament or meniscus surgery was the most common for knee meniscal and ligament injuries. Specifically, replacement arthroplasty usually involved total arthroplasty, and menisectomy was performed as either unilateral medial or unilateral lateral menisectomy. Patients who received surgical care were generally hospitalized.

Subcutaneous and intramuscular injections were performed in 19,722 knee OA cases and in 7390 meniscal and ligament injury cases, taking up approximately 40% of the cases of each disorder. In contrast, intra-articular injections were performed in 26,883 (55%) knee OA cases and in 549 (3%) meniscal and ligament injury cases. Upon hospitalization, subcutaneous and intramuscular injection rates increased to 83% and 65% for knee OA and meniscal and ligament injury patients, respectively, representing rates that were approximately twice those observed in outpatients. Intra-articular injections were administered to 55% of knee OA outpatients, but to only 14% of inpatients.

Of various physiotherapy modalities, superficial heat therapy, deep heat therapy, transcutaneous electrical nerve stimulation, and interferential current therapy were prescribed to about 25% of patients of either knee disorder; other physiotherapies were seldom used. Physiotherapy use was slightly more common for knee OA than meniscal and ligament injury patients. However, in hospitalized patients, exercise therapy was prescribed to 59% of knee OA and 27% of meniscal and ligament injury patients, which is significantly higher than the rates observed in ambulatory settings (Table 4).

**Table 4:** Usual care of knee osteoarthritis and knee meniscal and ligament injury, excluding prescription medication use

	Subtypes	Total				Inpatient				Outpatient			
		Knee OA		Knee meniscal and ligament injury		Knee OA		Knee meniscal and ligament injury		Knee OA		Knee meniscal and ligament injury	
		N = 48,321	%	N = 19,136	%	N = 3084	%	N = 2434	%	N = 48,000	%	N = 18,540	%
Surgery	Replacement Arthroplasty, Total Arthroplasty, knee	1466	3.03	1	0.01	1466	47.54	1	0.04	–	–	–	–
	Replacement Arthroplasty, Hemiarthroplasty, knee	89	0.18	–	–	89	2.89	–	–	–	–	–	–
	Revision of Replacement Arthroplasty, Total Arthroplasty, knee	26	0.05	–	–	26	0.84	–	–	–	–	–	–
	Revision of Replacement Arthroplasty, Hemiarthroplasty, knee	8	0.02	–	–	8	0.26	–	–	–	–	–	–
	Menisectomy, Medial or Lateral	182	0.38	586	3.06	182	5.90	584	23.99	–	–	2	0.01
	Menisectomy, Medial and Lateral	44	0.09	151	0.79	44	1.43	151	6.20	–	–	–	–
	Repair of Meniscus, Medial or Lateral	18	0.04	222	1.16	18	0.58	222	9.12	–	–	–	–
	Repair of Meniscus, Medial and Lateral	2	0.00	35	0.18	2	0.06	35	1.44	–	–	–	–
	Reconstruction of Cruciate Ligament	–	–	446	2.33	–	–	446	18.32	–	–	–	–
	Repair of Cruciate Ligament	1	0.00	11	0.06	1	0.03	11	0.45	–	–	–	–
Injection	Intraarticular Injection	26,883	55.63	334	1.75	427	13.85	107	4.40	26,695	55.61	296	1.60
	Subcutaneous or Intramuscular Injection	19,722	40.81	6457	33.74	2572	83.40	1775	72.93	18,265	38.05	5279	28.47
Physiotherapy	Superficial Heat Therapy	24,261	50.21	8774	45.85	1486	48.18	952	39.11	23,575	49.11	8319	44.87
	Deep Heat Therapy	22,128	45.79	8032	41.97	1285	41.67	863	35.46	21,511	44.81	7603	41.01
	Transcutaneous Electrical Nerve Stimulation	14,281	29.55	4804	25.10	969	31.42	572	23.50	13,736	28.62	4467	24.09
	Interferential Current Therapy	12,306	25.47	4195	21.92	980	31.78	563	23.13	11,698	24.37	3853	20.78
	Laser Therapy^a^	3423	7.08	1705	8.91	327	10.60	216	8.87	3172	6.61	1557	8.40
	Therapeutic Exercise^a^	2016	4.17	811	4.24	1806	58.56	652	26.79	612	1.28	381	2.06
	Simple Therapeutic Exercise	1712	3.54	673	3.52	434	14.07	174	7.15	1358	2.83	537	2.90
	Cold Therapy, Cold Pack	1119	2.32	785	4.10	423	13.72	195	8.01	774	1.61	647	3.49
	Myofascial Trigger Point Injection Therapy^b^	609	1.26	58	0.30	37	1.20	7	0.29	576	1.20	51	0.28

*OA* Osteoarthritis

^a^Simple rehabilitation treatments, which can be prescribed by specialists of rehabilitation medicine, orthopedic surgery, neurosurgery, neurology, general surgery, cardiovascular surgery, and anesthesiology

^b^Complex rehabilitation treatment, which can only be prescribed by specialists of rehabilitation medicine

### Medication use in usual care of knee OA and knee meniscal and ligament injuries

The medications, including both narcotics and non-narcotics, frequently used for knee disorders were organized according to 5th ATC levels. The most frequently used medication, for both knee OA and meniscal and ligament injury patients, was aceclofenac, which was administered to 20–30% of all knee disorder patients. Tramadol and diclofenac were also commonly prescribed, but their use was more pronounced in inpatients than in outpatients. In contrast, paracetamol was more frequently used among outpatients than among inpatients. Meloxicam and celecoxib were also largely used for knee OA patients, but their use was not evident in knee meniscal and ligament injury patients. Pethidine was the most commonly used narcotic analgesic, with 25% of knee OA inpatients receiving pethidine, although the overall prescription rate was low (Table 5).

**Table 5:** Medication prescribed for knee osteoarthritis and knee meniscal and ligament injury as assessed at the 5th Anatomical Therapeutic Chemical Classification System level

5th ATC level	Total				Inpatient				Outpatient			
	Knee OA		Knee meniscal and ligament injury		Knee OA		Knee meniscal and ligament injury		Knee OA		Knee meniscal and ligament injury	
	*N* = 48,321	%	*N* = 19,136	%	*N* = 3084	%	*N* = 2434	%	*N* = 48,000	%	*N* = 18,540	%
Aceclofenac	15,810	32.72	4961	25.92	840	27.24	858	35.25	15,357	31.99	4499	24.27
Tramadol	10,651	22.04	2982	15.58	1743	56.52	837	34.39	9364	19.51	2291	12.36
Diclofenac	9068	18.77	3688	19.27	1764	57.20	1132	46.51	7734	16.11	2832	15.28
Meloxicam	10,785	22.32	597	3.12	524	16.99	178	7.31	10,544	21.97	486	2.62
Loxoprofen sodium hydrate^a^	5598	11.59	3295	17.22	291	9.44	358	14.71	5375	11.20	3043	16.41
Tramadol, combinations	7082	14.66	1588	8.30	582	18.87	248	10.19	6741	14.04	1423	7.68
Talniflumate^a^	5273	10.91	3127	16.34	432	14.01	519	21.32	4944	10.30	2747	14.82
Paracetamol	4988	10.32	1435	7.50	843	27.33	364	14.95	4299	8.96	1133	6.11
Celecoxib	4791	9.91	134	0.70	634	20.56	43	1.77	4490	9.35	106	0.57
Piroxicam	2085	4.31	307	1.60	220	7.13	116	4.77	1908	3.98	200	1.08
Zaltoprofen^a^	1531	3.17	753	3.93	138	4.47	113	4.64	1432	2.98	666	3.59
Chlorphenesin carbamate^a^	1597	3.30	599	3.13	69	2.24	46	1.89	1565	3.26	568	3.06
Dexibuprofen	1105	2.29	509	2.66	36	1.17	23	0.94	1072	2.23	488	2.63
Ketorolac	777	1.61	279	1.46	633	20.53	230	9.45	152	0.32	49	0.26
Nabumetone	904	1.87	114	0.60	46	1.49	37	1.52	873	1.82	83	0.45
Pethidine^b^	858	1.78	151	0.79	843	27.33	145	5.96	18	0.04	7	0.04

*ATC* Anatomical Therapeutic Chemical, *OA* Osteoarthritis

^a^Chemical name of medicine with no corresponding 5th level ATC codes

^b^Narcotics; otherwise, non-narcotics

## Discussion

The total expenses, per-patient expenses, average days of care, and average number of visits were higher for inpatients than for outpatients, regardless of the specific type of knee disorder diagnosis (knee OA or meniscal and ligament injury). These results may be partly attributed to differences in disease and symptom severity as patients with more severe disease and/or symptoms may be more likely to undergo hospitalized care. Another possible explanation is that surgical interventions are more often performed in inpatient settings (Table 4). The current results support the view that surgical interventions take up a substantial portion of knee disorder-related costs. The incidence of knee OA was highest among females aged ≥50 years, which is consistent with a previous report by Arden et al. [20]. Conversely, knee meniscal and ligament injuries occurred more often among those aged <60 years. Although the incidence of these injuries was highest among children and adolescents, hospitalized patients amounted to only half of the outpatient sector in this age group, suggesting that physically active younger individuals may be more prone to injury but injuries may be milder.

The costs for procedures/surgeries, injections, visits (consultations), and hospitalizations comprised ≥75% of the total expenses for patients with either type of knee disorder. Although the large number of injection treatments is mainly responsible for this finding, it is worth note that the number of surgery and hospitalization cases was relatively small compared to the total costs. It can be carefully inferred that performing surgical operations in inpatient settings is likely to drastically increase per-patient expenses. Injection treatments constituted the second largest expense in knee OA patients, and hospitalization was the 2nd largest expense for knee meniscal and ligament injury patients. To ease the socioeconomic burden of these disorders (i.e., total expenses and per-patient expenses), studies should be conducted, from the policymakers’ perspective, on appropriate practice guidelines and adequate compensation costs, especially regarding surgical interventions, injection treatments, and hospitalization.

The types of surgical interventions performed in the two diagnostic groups were highly disparate, with replacement arthroplasty being performed most frequently in cases of knee OA, and meniscal and ligament operations (i.e., injured menisci or ligaments) being most common in cases of knee meniscal and ligament injury. Subcutaneous and intramuscular injection treatments were conducted in 19,722 knee OA cases and in 7390 knee meniscal and ligament injury cases, each comprising about 40% of all cases. However, the percentages of patients receiving subcutaneous and intramuscular injections rose to 83% in knee OA inpatients and to 65% in meniscal and ligament injury inpatients. Although intra-articular injections were frequently employed for knee OA outpatients, they were seldom used in patients with knee meniscal and ligament injury.

Heat therapy and electrostimulation are two types of physiotherapy that may be considered typical care for knee OA and meniscal and ligament injuries. More than 58% of hospitalized knee OA patients were prescribed therapeutic exercise, which was more common than superficial heat therapy. Therapeutic exercise is speculated to be mainly used as a means of post-surgical rehabilitation. Laser therapy and therapeutic exercise were considered simple rehabilitation treatment methods; myofascial trigger point injection, a complex rehabilitation method; and the other subtypes to be basic physiotherapies. Basic physiotherapy can be prescribed by all physicians without restriction as opposed to simple and complex rehabilitation therapies which can only be prescribed by relevant specialists. Therefore, limitations in the ability to prescribe laser therapy, therapeutic exercise, and myofascial trigger point injection treatments should be considered when interpreting these results.

Diclofenac is a non-steroidal anti-inflammatory drug (NSAID) that is widely used as first-line therapy for chronic inflammatory states including OA. However, prolonged use of diclofenac has been associated with gastrointestinal adverse events such as bleeding, ulcers, and perforations in severe cases. Aceclofenac, which has a similar chemical structure, was developed to address such complications and has been proven to be a safer alternative [21]. Aceclofenac was shown to be used more frequently than diclofenac in this study, possibly because it exhibits fewer side effects. Diclofenac tended to be more frequently administered in inpatient settings where adverse event monitoring is easier.

Tramadol is an opioid-like analgesic that affects the central nervous system (CNS) [22]. Tramadol, a μ-opioid receptor agonist, exerts nociceptive effects by increasing serotonin and noradrenaline levels in the CNS [23]. As the mechanisms of action differ between tramadol and NSAIDs, tramadol can be effectively used in patients with pain not responding adequately to NSAID treatment [24]. The present study results revealed that tramadol was the second most commonly prescribed drug. Moreover, tramadol combinations were the sixth most common prescription, under a separate ATC code, and taken together, the number of tramadol prescriptions was nearly as high as those for aceclofenac.

Paracetamol, also known as acetaminophen, is recommended as a first-line analgesic for knee OA patients by the European League against Rheumatism, American College of Rheumatology (ACR), and Osteoarthritis Research Society International. Long-term use of paracetamol is also preferred over other medications [25]. Although the analgesic effect of paracetamol is relatively weak compared to that of NSAIDs or COX-2 selective inhibitors, it is widely used as it is better tolerated [26]. However, paracetamol is often implicated in drug-induced liver injuries with approximately 30,000 patients being hospitalized annually for paracetamol-related liver injuries in the United States [27]. Nevertheless, paracetamol hepatotoxicity has been reported to be minimized by avoiding overdosing [28, 29].

One strength of classifying drugs according to 5th ATC levels is that complete identification of specific chemicals is possible, while limitations include the fact that identification and recognition of prescription patterns is not easy. The most frequently prescribed drug substances are listed in Table 5. Complete listings of prescribed drugs are organized by non-narcotics and narcotics in Additional file 3: Table S3 and Additional file 4: Table S4. At the 4th ATC level, the non-narcotic and narcotic drugs consisted of 18 and 4 subgroups, respectively (Additional file 5: Table S5). In order of prescription frequency, acetic acid derivatives and related substances, other opioids, propionic acid derivatives, oxicams, other anti-inflammatory and anti-rheumatic agents, and non-steroids were the most frequently used drugs in the non-narcotic analgesic subgroups, and phenylpiperidine derivatives, natural opium alkaloids, opioid anesthetics, and opium alkaloids and their derivatives were the most frequently prescribed drugs from the narcotic analgesic subgroups.

Investigations of medical service use at a national level hold heightened significance in that various disparities exist between evidence-based medicine and the actual practice selected for knee disorders in real world settings. For example, while use of meniscectomy is similarly high in Korea as in the U.S., a recent high-quality RCT reported that its effects did not surpass that of sham controls [30]. Similarly, although total knee replacement (TKR) is the most common surgical intervention for knee OA in Korea and is performed in 670,000 new cases in the U.S. annually, comparison of TKR and 12 weeks of conservative treatment found that while TKR resulted in significant differences in pain reduction, it also entailed serious complications, and most of the conservative treatment group exhibited meaningful improvement without surgery [31]. Moreover, intra-articular triamcinolone injections, which are commonly used in knee OA patients, failed to provide pain relief over saline, and were associated with a significantly larger decrease in joint cartilage [32].

Study limitations include the following: While the study analyses were based on codes filed in the national claims database, the level of accuracy of the diagnosing process itself could not be verified and is beyond the scope of this study. For example, a physician may have reached a diagnosis not conforming to such well-established diagnostic standards as proposed by the ACR [33]. In addition, outpatient medication intake could only be assessed from database prescription records rather than actual medication intake records. Regarding terminology, although the term incidence was used throughout the manuscript to indicate new cases within the index period, readers should take into account that the incidence was limited to new symptomatic cases that received diagnosis and treatment through visits to medical institutions for medical service use. Moreover, the current database does not contain information regarding treatments and medications not covered by National Health Insurance or over-the-counter medication. While other studies using claims data carry similar limitations, these uncertainties need to be given due consideration when interpreting these results.

Records of medical services, including surgeries, injections, physiotherapy, and analgesic use, could not be solely attributed to knee disorders if the patient had coexisting conditions and was coded with different subsets of disease codes. Although codes that were clearly non-knee-associated were excluded, such consolidations may be an additional limitation of this study, as it may have introduced unintentional selection bias. Also of note is that both conventional and traditional Korean medicine (TKM) are recognized by Korean medical and legal regulatory bodies in a dual medical system and are covered by National Health Insurance. Many patients seek acupuncture and pharmacopuncture for treatment of musculoskeletal disorders. In a previous report on TKM care and low back pain in Korea using 2011 HIRA-NPS data, TKM use was highest (28.8%) in patients with low back pain [34]. Unfortunately, the 2014 dataset currently available to the public does not include records on TKM use, limiting the present study results to conventional medicine services, and precluding a more comprehensive and complete assessment of knee disorder treatments in Korea.

## Conclusions

This study analyzed HIRA-NPS data to investigate the current incidence of knee disorders and clinical practice patterns for knee OA and knee meniscal and ligament injuries. The medical expense analysis (i.e., amounts and distribution) may provide further information to policymakers. Further, the detailed description of injection, physiotherapy, and analgesic use may prove valuable for researchers and practitioners seeking to understand the constituents of usual care in actual clinical practice. Additional research is warranted, particularly regarding treatment codes (e.g., M22 and M23) not covered in the present study. Also, the HIRA-NPS dataset only contains billing records for a single calendar year, rendering longitudinal studies with time series analyses of natural history and causal relationships impossible; national cohort data extracted from national health insurance claims data [35] may be analyzed for such purposes in future studies.

## Additional files

Additional file 1: Table S1. Diagnostic codes of knee disorders following the Korean Standard Classification of Diseases, 6th revision (KCD-6) adapted from the International Classification of Diseases, 10th revision. (ICD-10) (DOCX 20 kb)

Additional file 2: Table S2. Definition of medical care for knee disorders from given codes. (DOCX 18 kb)

Additional file 3: Table S3. Non-narcotic medications in knee osteoarthritis and knee meniscal and ligament injury as assessed at the 5th Anatomical Therapeutic Chemical Classification System level. (DOCX 30 kb)

Additional file 4: Table S4. Narcotic medications in knee osteoarthritis and knee meniscal and ligament injury as assessed at the 5th Anatomical Therapeutic Chemical Classification System level. (DOCX 19 kb)

Additional file 5: Table S5. Total medications in knee osteoarthritis and knee meniscal and ligament injury as assessed at the 4th Anatomical Therapeutic Chemical Classification System level. (DOCX 23 kb)

## Abbreviations

ACL: Anterior cruciate ligament
ACR: American College of Rheumatology
ATC: Anatomical Therapeutic Chemical
CNS: Central nervous system
COX-2: Cyclooxygenase-2
EM: Emergency medicine
HIRA: Korean Health Insurance Review and Assessment Service
ICD: International Classification of Diseases
NHIS: National Health Insurance Service
NPS: National Patient Sample
NSAID: Non-steroidal anti-inflammatory drug
OA: Osteoarthritis
TKM: Traditional Korean medicine
TKR: Total knee replacement
