# Supplementary material for: Analysis of medical service use of knee osteoarthritis and knee meniscal and ligament injuries in Korea: a cross-sectional study of national patient sample data
Source: BMC Musculoskelet Disord. 2017 Nov 10;18:438. doi: 10.1186/s12891-017-1795-7 (PMC5681826; doi:10.1186/s12891-017-1795-7)
Supplement: Supplementary file 1 — Diagnostic codes of knee disorders following the Korean Standard Classification of Diseases, 6th revision (KCD-6) adapted from the International Classification of Diseases, 10th revision. (ICD-10) (DOCX 20 kb) [file 12891_2017_1795_MOESM1_ESM.docx]

**Supplementary Table 1** Diagnostic codes of knee disorders following the Korean Standard Classification of Diseases, 6^th^ revision (KCD-6) adapted from the International Classification of Diseases, 10^th^ revision (ICD-10)

| KCD code | KCD diagnosis | Number of patients | Total expense^*^ | Days of treatment^†^ | Number of visits^‡^ |
| --- | --- | --- | --- | --- | --- |
| M17 | Gonarthrosis[arthrosis of knee] | 48,321 | 507,579.3 | 11.0 | 9.1 |
| M170 | Primary gonarthrosis, bilateral | 31,211 | 480550.3 | 9.2 | 8.0 |
| M171 | Other primary gonarthrosis | 16,911 | 324466.6 | 7.1 | 6.2 |
| M172 | Post-traumatic gonarthrosis, bilateral | 84 | 344523.7 | 6.7 | 5.6 |
| M173 | Other post-traumatic gonarthrosis | 341 | 197571.2 | 5.8 | 5.2 |
| M174 | Other secondary gonarthrosis, bilateral | 769 | 228702.5 | 6.1 | 5.5 |
| M175 | Other secondary gonarthrosis | 742 | 238835.7 | 5.7 | 5.3 |
| M179 | Gonarthrosis, unspecified | 13,324 | 268819.0 | 8.3 | 5.7 |
| M22 | Disorsers of patella | 3,087 | 102,504.3 | 3.2 | 2.8 |
| M220 | Recurrent dislocation of patella | 13 | 721502.31 | 9.6 | 5.4 |
| M221 | Recurrent subluxation of patella | 13 | 489586.15 | 7.9 | 4.6 |
| M222 | Patellofemoral disorders | 288 | 68626.15 | 2.3 | 2.1 |
| M223 | Other derangements of patella | 55 | 65254.18 | 2.3 | 1.9 |
| M224 | Chondromalacia patellae | 2,670 | 98326.1 | 3.2 | 2.8 |
| M228 | Other disorders of patella | 32 | 291720 | 6.8 | 6.3 |
| M229 | Disorder of patella, unspecified | 44 | 124259.32 | 3.8 | 3.6 |
| M23 | Internal derangement of knee | 7,103 | 457,057.8 | 7.8 | 5.5 |
| M230 | Cystic meniscus | 33 | 539046.06 | 6.5 | 3.9 |
| M231 | Discoid meniscus(congenital) | 116 | 689981.12 | 10.4 | 6.6 |
| M232 | Derangement of meniscus due to old tear or injury | 1,960 | 932661.17 | 13.5 | 8.5 |
| M233 | Other meniscus derangements | 588 | 526386.85 | 8.9 | 5.9 |
| M234 | Loose body in knee | 16 | 507051.25 | 8.7 | 5.9 |
| M235 | Chronic instability of knee | 97 | 756865.15 | 7.9 | 5.7 |
| M236 | Other spontaneous disruption of ligament(s) of knee | 72 | 652648.06 | 10.0 | 6.5 |
| M238 | Other internal derangement of knee | 2,276 | 223604.61 | 4.8 | 3.8 |
| M239 | Internal derangement of knee, unspecified | 2,704 | 138047.4 | 3.7 | 3.0 |
| S83 | Dislocation, sprain and strain of joints and ligaments of knee | 19,136 | 303,133.4 | 5.8 | 4.7 |
| S830 | Dislocation of patella | 56 | 735,327.2 | 11.9 | 7.6 |
| S831 | Dislocation of knee | 28 | 339,313.8 | 5.1 | 3.3 |
| S832 | Tear of meniscus, current | 2,247 | 771,334.7 | 12.4 | 8.6 |
| S833 | Tear of articular cartilage of knee, current | 234 | 492,268.6 | 8.9 | 7.2 |
| S834 | Sprain and strain involving (fibular)(tibial) collateral ligament of knee | 3,298 | 177,852.5 | 4.7 | 4.2 |
| S835 | Sprain and strain involving (anterior)(posterior) cruciate ligament of knee | 1,530 | 1,285,459.7 | 15.8 | 11.5 |
| S836 | Sprain and strain of other and unspecified parts of knee | 13,193 | 77,474.1 | 2.9 | 2.6 |
| S837 | Injury to multiple structures of knee | 358 | 379,336.2 | 7.1 | 5.8 |

^*^Displayed in KRW; 1 USD=1,104 KRW (as of September 30^th^, 2016)

^†^The total days of treatment indicated in the claims statement including drug prescription days without medical treatment.

^‡^The number of outpatient visits or the number of inpatient care days of the patient indicated in the claims statement.

KCD, Korean Standard Classification of Diseases
