# Supplementary material for: Analysis of medical service use of knee osteoarthritis and knee meniscal and ligament injuries in Korea: a cross-sectional study of national patient sample data
Source: BMC Musculoskelet Disord. 2017 Nov 10;18:438. doi: 10.1186/s12891-017-1795-7 (PMC5681826; doi:10.1186/s12891-017-1795-7)
Supplement: Supplementary file 2 — Definition of medical care for knee disorders from given codes. (DOCX 18 kb) [file 12891_2017_1795_MOESM2_ESM.docx]

**Supplementary Table 2** Definition of medical care for knee disorders from given codes

| Code | Code name | Care name |
| --- | --- | --- |
| N2072 | Replacement Arthroplasty, Total Arthroplasty, Knee | Replacement Arthroplasty, Total Arthroplasty, knee |
| N2077 | Replacement Arthroplasty, Total Arthroplasty, Knee, Complex |  |
| N2712 | Replacement Arthroplasty, Hemiarthroplasty, Knee | Replacement Arthroplasty, Hemiarthroplasty, knee |
| N2717 | Replacement Arthroplasty, Hemiarthroplasty, Knee, Complex |  |
| N3712 | Revision of Replacement Arthroplasty, Total Arthroplasty, Knee | Revision of Replacement Arthroplasty, Total Arthroplasty, knee |
| N3717 | Revision of Replacement Arthroplasty, Total Arthroplasty, Knee, Complex |  |
| N3722 | Revision of Replacement Arthroplasty, Total Arthroplasty, Knee, Resection of previous artificial implant |  |
| N3727 | Revision of Replacement Arthroplasty, Total Arthroplasty, Knee, Resection of previous artificial implant, Complex |  |
| N4712 | Revision of Replacement Arthroplasty, Hemiarthroplasty, Knee | Revision of Replacement Arthroplasty, Hemiarthroplasty, knee |
| N4717 | Revision of Replacement Arthroplasty, Hemiarthroplasty, Knee, Complex |  |
| N4722 | Revision of Replacement Arthroplasty, Hemiarthroplasty, Knee, Resection of previous artificial implant |  |
| N4727 | Revision of Replacement Arthroplasty, Hemiarthroplasty, Knee, Resection of previous artificial implant, Complex |  |
| N0821 | Menisectomy, Medial or Lateral | Menisectomy, Medial or Lateral |
| N0826 | Menisectomy, Medial or Lateral, Complex |  |
| N0822 | Menisectomy, Medial and Lateral | Menisectomy, Medial and Lateral |
| N0827 | Menisectomy, Medial and Lateral, Complex |  |
| N0823 | Repair of Meniscus, Medial or Lateral | Repair of Meniscus, Medial or Lateral |
| N0828 | Repair of Meniscus, Medial or Lateral, Complex |  |
| N0824 | Repair of Meniscus, Medial and Lateral | Repair of Meniscus, Medial and Lateral |
| N0829 | Repair of Meniscus, Medial and Lateral, Complex |  |
| N0880 | Reconstruction of Cruciate Ligament | Reconstruction of Cruciate Ligament |
| N0881 | Reconstruction of Cruciate Ligament, Complex |  |
| N0890 | Repair of Cruciate Ligament | Repair of Cruciate Ligament |
| KK090 | Intraarticular Injection | Intraarticular Injection |
| KK010 | Subcutaneous or Intramuscular Injection | Subcutaneous or Intramuscular Injection |
| MM010 | Superficial Heat Therapy | Superficial Heat Therapy |
| MM015 | Superficial Heat Therapy (On the same day with MM020 Deep Heat Therapy) |  |
| MM020 | Deep Heat Therapy | Deep Heat Therapy |
| MM070 | Transcutaneous Electrical Nerve Stimulation | Transcutaneous Electrical Nerve Stimulation |
| MM080 | Interferential Current Therapy | Interferential Current Therapy |
| MM085 | Laser Therapy | Laser Therapy |
| MM102 | Therapeutic Exercise | Therapeutic Exercise |
| MM101 | Simple Therapeutic Exercise | Simple Therapeutic Exercise |
| MM011 | Cold Therapy | Cold Therapy |
| MM131 | Myofascial Trigger Point Injection Therapy | Myofascial Trigger Point Injection Therapy |
