# Supplementary material for: Analysis of medical service use of knee osteoarthritis and knee meniscal and ligament injuries in Korea: a cross-sectional study of national patient sample data
Source: BMC Musculoskelet Disord. 2017 Nov 10;18:438. doi: 10.1186/s12891-017-1795-7 (PMC5681826; doi:10.1186/s12891-017-1795-7)
Supplement: Supplementary file 3 — Non-narcotic medications in knee osteoarthritis and knee meniscal and ligament injury as assessed at the 5th Anatomical Therapeutic Chemical Classification System level. (DOCX 30 kb) [file 12891_2017_1795_MOESM3_ESM.docx]

**Supplementary Table 3** Non-narcotic medications in knee osteoarthritis and knee meniscal and ligament injury as assessed at the 5^th^ Anatomical Therapeutic Chemical Classification System level

| 5^th^ ATC level | Total | | | | Inpatient | | | | Outpatient | | | |
| --- | --- | --- | --- | --- | --- | --- | --- | --- | --- | --- | --- | --- |
|  | **Knee OA** | | **Knee meniscal and ligament injury** | | **Knee OA** | | **Knee meniscal and ligament injury** | | **Knee OA** | | **Knee meniscal and ligament injury** | |
|  | N=48,321 | % | N=19,136 | % | N=3,084 | % | N=2,434 | % | N=48,000 | % | N=18,540 | % |
| Aceclofenac | 15,810 | 32.72 | 4,961 | 25.92 | 840 | 27.24 | 858 | 35.25 | 15,357 | 31.99 | 4,499 | 24.27 |
| Tramadol | 10,651 | 22.04 | 2,982 | 15.58 | 1,743 | 56.52 | 837 | 34.39 | 9,364 | 19.51 | 2,291 | 12.36 |
| Diclofenac | 9,068 | 18.77 | 3,688 | 19.27 | 1,764 | 57.20 | 1,132 | 46.51 | 7,734 | 16.11 | 2,832 | 15.28 |
| Meloxicam | 10,785 | 22.32 | 597 | 3.12 | 524 | 16.99 | 178 | 7.31 | 10,544 | 21.97 | 486 | 2.62 |
| Loxoprofen sodium hydrate^*^ | 5,598 | 11.59 | 3,295 | 17.22 | 291 | 9.44 | 358 | 14.71 | 5,375 | 11.20 | 3,043 | 16.41 |
| Tramadol, combinations | 7,082 | 14.66 | 1,588 | 8.30 | 582 | 18.87 | 248 | 10.19 | 6,741 | 14.04 | 1,423 | 7.68 |
| Talniflumate^*^ | 5,273 | 10.91 | 3,127 | 16.34 | 432 | 14.01 | 519 | 21.32 | 4,944 | 10.30 | 2,747 | 14.82 |
| Paracetamol | 4,988 | 10.32 | 1,435 | 7.50 | 843 | 27.33 | 364 | 14.95 | 4,299 | 8.96 | 1,133 | 6.11 |
| Celecoxib | 4,791 | 9.91 | 134 | 0.70 | 634 | 20.56 | 43 | 1.77 | 4,490 | 9.35 | 106 | 0.57 |
| Piroxicam | 2,085 | 4.31 | 307 | 1.60 | 220 | 7.13 | 116 | 4.77 | 1,908 | 3.98 | 200 | 1.08 |
| Zaltoprofen^*^ | 1,531 | 3.17 | 753 | 3.93 | 138 | 4.47 | 113 | 4.64 | 1,432 | 2.98 | 666 | 3.59 |
| Chlorphenesin carbamate^*^ | 1,597 | 3.30 | 599 | 3.13 | 69 | 2.24 | 46 | 1.89 | 1,565 | 3.26 | 568 | 3.06 |
| Dexibuprofen | 1,105 | 2.29 | 509 | 2.66 | 36 | 1.17 | 23 | 0.94 | 1,072 | 2.23 | 488 | 2.63 |
| Ketorolac | 777 | 1.61 | 279 | 1.46 | 633 | 20.53 | 230 | 9.45 | 152 | 0.32 | 49 | 0.26 |
| Nabumetone | 904 | 1.87 | 114 | 0.60 | 46 | 1.49 | 37 | 1.52 | 873 | 1.82 | 83 | 0.45 |
| Pelubiprofen^*^ | 877 | 1.81 | 117 | 0.61 | 36 | 1.17 | 36 | 1.48 | 849 | 1.77 | 89 | 0.48 |
| Ibuprofen | 245 | 0.51 | 321 | 1.68 | 11 | 0.36 | 21 | 0.86 | 235 | 0.49 | 305 | 1.65 |
| Etodolac | 298 | 0.62 | 192 | 1.00 | 9 | 0.29 | 14 | 0.58 | 290 | 0.60 | 182 | 0.98 |
| Propofol | 275 | 0.57 | 194 | 1.01 | 274 | 8.88 | 194 | 7.97 | 1 | 0.00 | - | - |
| Morniflumate | 240 | 0.50 | 79 | 0.41 | 12 | 0.39 | 12 | 0.49 | 228 | 0.48 | 67 | 0.36 |
| Mefenamic acid | 220 | 0.46 | 72 | 0.38 | 33 | 1.07 | 14 | 0.58 | 187 | 0.39 | 60 | 0.32 |
| Naproxen | 203 | 0.42 | 46 | 0.24 | 9 | 0.29 | 5 | 0.21 | 196 | 0.41 | 41 | 0.22 |
| Propacetamol | 122 | 0.25 | 70 | 0.37 | 119 | 3.86 | 66 | 2.71 | 3 | 0.01 | 4 | 0.02 |
| Cyclobenzaprine | 81 | 0.17 | 32 | 0.17 | 8 | 0.26 | 5 | 0.21 | 75 | 0.16 | 28 | 0.15 |
| Proglumetacin | 79 | 0.16 | 25 | 0.13 | - | - | 2 | 0.08 | 79 | 0.16 | 23 | 0.12 |
| Nimesulide | 71 | 0.15 | 24 | 0.13 | 11 | 0.36 | 2 | 0.08 | 62 | 0.13 | 22 | 0.12 |
| Ketoprofen, combinations | 73 | 0.15 | 9 | 0.05 | 21 | 0.68 | 2 | 0.08 | 52 | 0.11 | 7 | 0.04 |
| Acetylsalicylic acid | 57 | 0.12 | 11 | 0.06 | 40 | 1.30 | 6 | 0.25 | 19 | 0.04 | 5 | 0.03 |
| Sulindac | 53 | 0.11 | 6 | 0.03 | 2 | 0.06 | 1 | 0.04 | 52 | 0.11 | 5 | 0.03 |
| Hydroxocobalamin | 34 | 0.07 | 22 | 0.11 | 20 | 0.65 | 21 | 0.86 | 14 | 0.03 | 1 | 0.01 |
| Dexamethasone | 44 | 0.09 | 1 | 0.01 | - | - | - | - | 44 | 0.09 | 1 | 0.01 |
| Nalbufine | 25 | 0.05 | 14 | 0.07 | 25 | 0.81 | 14 | 0.58 | - | - | - | - |
| Lornoxicam | 35 | 0.07 | - | - | - | - | - | - | 35 | 0.07 | - | - |
| Butorphanol | 28 | 0.06 | 5 | 0.03 | 28 | 0.91 | 5 | 0.21 | - | - | - | - |
| Acemetacin | 29 | 0.06 | 1 | 0.01 | - | - | - | - | 29 | 0.06 | 1 | 0.01 |
| Thiopental | 14 | 0.03 | 6 | 0.03 | 14 | 0.45 | 6 | 0.25 | - | - | - | - |
| Diclofenac, combinations | 15 | 0.03 | - | - | - | - | - | - | 15 | 0.03 | - | - |
| Ketamine | 11 | 0.02 | 2 | 0.01 | 11 | 0.36 | 2 | 0.08 | - | - | - | - |
| Oxaprozin | 7 | 0.01 | - | - | - | - | - | - | 7 | 0.01 | - | - |
| Acetylsalicylic acid | 6 | 0.01 | - | - | - | - | - | - | 6 | 0.01 | - | - |
| Tiaprofenic acid | 5 | 0.01 | - | - | - | - | - | - | 5 | 0.01 | - | - |
| Salsalate | 3 | 0.01 | 1 | 0.01 | 3 | 0.10 | 1 | 0.04 | - | - | - | - |
| Etomidate | 1 | 0.00 | - | - | - | - | - | - | 1 | 0.00 | - | - |
| Pyrazinobutazone^*^ | 1 | 0.00 | - | - | - | - | - | - | 1 | 0.00 | - | - |
| Almotriptan | - | - | 1 | 0.01 | - | - | - | - | - | - | 1 | 0.01 |
| Pranoprofen^*^ | - | - | 1 | 0.01 | - | - | - | - | - | - | 1 | 0.01 |

^*^Chemical name of medicine with no corresponding 5^th^ level ATC codes

ATC, Anatomical Therapeutic Chemical; OA, Osteoarthritis
