# Supplementary material for: Analysis of medical service use of knee osteoarthritis and knee meniscal and ligament injuries in Korea: a cross-sectional study of national patient sample data
Source: BMC Musculoskelet Disord. 2017 Nov 10;18:438. doi: 10.1186/s12891-017-1795-7 (PMC5681826; doi:10.1186/s12891-017-1795-7)
Supplement: Supplementary file 4 — Narcotic medications in knee osteoarthritis and knee meniscal and ligament injury as assessed at the 5th Anatomical Therapeutic Chemical Classification System level. (DOCX 19 kb) [file 12891_2017_1795_MOESM4_ESM.docx]

**Supplementary Table 4** Narcotic medications in knee osteoarthritis and knee meniscal and ligament injury as assessed at the 5^th^ Anatomical Therapeutic Chemical Classification System level

| 5^th^ ATC level | Total | | | | Inpatient | | | | Outpatient | | | |
| --- | --- | --- | --- | --- | --- | --- | --- | --- | --- | --- | --- | --- |
|  | **Knee OA** | | **Knee meniscal and ligament injury** | | **Knee OA** | | **Knee meniscal and ligament injury** | | **Knee OA** | | **Knee meniscal and ligament injury** | |
|  | N=48,321 | % | N=19,136 | % | N=3,084 | % | N=2,434 | % | N=48,000 | % | N=18,540 | % |
| Pethidine | 858 | 1.78 | 151 | 0.79 | 843 | 27.33 | 145 | 5.96 | 18 | 0.04 | 7 | 0.04 |
| Fentanyl | 531 | 1.10 | 226 | 1.18 | 531 | 17.22 | 226 | 9.29 | - | - | - | - |
| Morphine | 510 | 1.06 | 29 | 0.15 | 495 | 16.05 | 28 | 1.15 | 24 | 0.05 | 1 | 0.01 |
| Codeine, combinations  (excluding psycholeptics) | 268 | 0.55 | 38 | 0.20 | 146 | 4.73 | 29 | 1.19 | 167 | 0.35 | 11 | 0.06 |
| Oxycodone | 150 | 0.31 | 25 | 0.13 | 140 | 4.54 | 23 | 0.94 | 24 | 0.05 | 3 | 0.02 |
| Remifentanil | 109 | 0.23 | 64 | 0.33 | 109 | 3.53 | 64 | 2.63 | - | - | - | - |
| Hydromorphone | 87 | 0.18 | 16 | 0.08 | 74 | 2.40 | 15 | 0.62 | 19 | 0.04 | 1 | 0.01 |
| Codeine | 23 | 0.05 | 5 | 0.03 | 16 | 0.52 | 3 | 0.12 | 7 | 0.01 | 2 | 0.01 |
| Alfentanil | 5 | 0.01 | 2 | 0.01 | 5 | 0.16 | 2 | 0.08 | - | - | - | - |
| Sufentanil | 2 | 0.00 | 1 | 0.01 | 2 | 0.06 | 1 | 0.04 | - | - | - | - |

ATC, Anatomical Therapeutic Chemical; OA, Osteoarthritis
