# Supplementary material for: Analysis of medical service use of knee osteoarthritis and knee meniscal and ligament injuries in Korea: a cross-sectional study of national patient sample data
Source: BMC Musculoskelet Disord. 2017 Nov 10;18:438. doi: 10.1186/s12891-017-1795-7 (PMC5681826; doi:10.1186/s12891-017-1795-7)
Supplement: Supplementary file 5 — Total medications in knee osteoarthritis and knee meniscal and ligament injury as assessed at the 4th Anatomical Therapeutic Chemical Classification System level. (DOCX 23 kb) [file 12891_2017_1795_MOESM5_ESM.docx]

**Supplementary Table 5** Total medications in knee osteoarthritis and knee meniscal and ligament injury as assessed at the 4^th^ Anatomical Therapeutic Chemical Classification System level

|  | 4^th^ ATC level | Total | | | | Inpatient | | | | Outpatient | | | |
| --- | --- | --- | --- | --- | --- | --- | --- | --- | --- | --- | --- | --- | --- |
|  |  | **Knee OA** | | **Knee meniscal and ligament injury** | | **Knee OA** | | **Knee meniscal and ligament injury** | | **Knee OA** | | **Knee meniscal and ligament injury** | |
|  |  | N=48,321 | % | N=19,136 | % | N=3,084 | % | N=2,434 | % | N=48,000 | % | N=18,540 | % |
| Non-narcotics | Acetic acid derivatives and related substances | 21,288 | 44.06 | 7,204 | 37.65 | 2,268 | 73.54 | 1,659 | 68.16 | 20,124 | 41.93 | 6,346 | 34.23 |
|  | Other opioids* | 15,192 | 31.44 | 4,096 | 21.40 | 1,963 | 63.65 | 996 | 40.92 | 14,029 | 29.23 | 3,376 | 18.21 |
|  | Propionic acid derivatives | 8,995 | 18.62 | 4,859 | 25.39 | 524 | 16.99 | 546 | 22.43 | 8,628 | 17.98 | 4,487 | 24.20 |
|  | Oxicams | 12,220 | 25.29 | 857 | 4.48 | 719 | 23.31 | 282 | 11.59 | 11,869 | 24.73 | 660 | 3.56 |
|  | Other antiinflammatory and antirheumatic agents, non-steroids | 6,354 | 13.15 | 3,297 | 17.23 | 500 | 16.21 | 568 | 23.34 | 5,996 | 12.49 | 2,892 | 15.60 |
|  | Anilides | 5,061 | 10.47 | 1,481 | 7.74 | 923 | 29.93 | 412 | 16.93 | 4,301 | 8.96 | 1,137 | 6.13 |
|  | Coxibs | 4,791 | 9.91 | 134 | 0.70 | 634 | 20.56 | 43 | 1.77 | 4,490 | 9.35 | 106 | 0.57 |
|  | Other centrally acting agents | 1,674 | 3.46 | 630 | 3.29 | 77 | 2.50 | 51 | 2.10 | 1,636 | 3.41 | 595 | 3.21 |
|  | Other general anesthetics | 286 | 0.59 | 196 | 1.02 | 285 | 9.24 | 196 | 8.05 | 1 | 0.00 | - | - |
|  | Fenamates | 220 | 0.46 | 72 | 0.38 | 33 | 1.07 | 14 | 0.58 | 187 | 0.39 | 60 | 0.32 |
|  | Morphinan derivatives | 53 | 0.11 | 19 | 0.10 | 53 | 1.72 | 19 | 0.78 | - | - | - | - |
|  | Salicylic acid and derivatives | 57 | 0.12 | 11 | 0.06 | 40 | 1.30 | 6 | 0.25 | 19 | 0.04 | 5 | 0.03 |
|  | Other nervous system drugs | 34 | 0.07 | 22 | 0.11 | 20 | 0.65 | 21 | 0.86 | 14 | 0.03 | 1 | 0.01 |
|  | Glucocorticoids | 44 | 0.09 | 1 | 0.01 | - | - | - | - | 44 | 0.09 | 1 | 0.01 |
|  | Barbiturates, plain | 14 | 0.03 | 6 | 0.03 | 14 | 0.45 | 6 | 0.25 | - | - | - | - |
|  | Platelet aggregation inhibitors excl. heparin | 4 | 0.01 | 2 | 0.01 | - | - | - | - | 4 | 0.01 | 2 | 0.01 |
|  | Butylpyrazolidines | 1 | 0.00 | - | - | - | - | - | - | 1 | 0.00 | - | - |
|  | Selective serotonin (5HT1) agonists | 1 | 0.00 | - | - | - | - | - | - | 1 | 0.00 | - | - |
| Narcotics | Phenylpiperidine derivatives | 858 | 1.78 | 151 | 0.79 | 843 | 27.33 | 145 | 5.96 | 18 | 0.04 | 7 | 0.04 |
|  | Natural opium alkaloids | 870 | 1.80 | 97 | 0.51 | 719 | 23.31 | 85 | 3.49 | 231 | 0.48 | 16 | 0.09 |
|  | Opioid anesthetics | 604 | 1.25 | 275 | 1.44 | 604 | 19.58 | 275 | 11.30 | - | - | - | - |
|  | Opium alkaloids and derivatives | 23 | 0.05 | 5 | 0.03 | 16 | 0.52 | 3 | 0.12 | 7 | 0.01 | 2 | 0.01 |

*Though labeled ‘other opioids’, 4^th^ ATC level other opioids include both narcotics and non-narcotics.

ATC, Anatomical Therapeutic Chemical; OA, Osteoarthritis
